# Expression of Concern for AIP1 functions as an endogenous inhibitor of VEGFR2-mediated signaling and inflammatory angiogenesis in mice

**DOI:** 10.1172/JCI198646

**Published:** 2025-09-16

**Authors:** Haifeng Zhang, Yun He, Shengchuan Dai, Zhe Xu, Yan Luo, Ting Wan, Dianhong Luo, Dennis Jones, Shibo Tang, Hong Chen, William C. Sessa, Wang Min

Original citation: *J Clin Invest*. 2008;118(12):3904-3916. https://doi.org/10.1172/JCI36168

Citation for this expression of concern: *J Clin Invest*. 2025;135(18):e198646. https://doi.org/10.1172/JCI198646

The authors recently became aware of errors in the Western blot data presented in Figures 6A and 3F and Supplemental Figure 6B, which included duplicated blot images that were used to represent distinct samples. Given the time that has elapsed since publication, some of the primary data related to these figures is no longer available. As we are unable to evaluate the integrity of all of these figure panels, the Editors are publishing this notice to alert readers to the concern.

## Version 1 09/16/2025

Electronic publication

